# Lipoprotein Lipase Inhibitor, Nordihydroguaiaretic Acid, Aggravates Metabolic Phenotypes and Alters HDL Particle Size in the Western Diet-Fed db/db Mice

**DOI:** 10.3390/ijms20123057

**Published:** 2019-06-22

**Authors:** Inhae Kang, Miyoung Park, Soo Jin Yang, Myoungsook Lee

**Affiliations:** 1 Department of Food Science and Nutrition, Jeju National University, Jeju 63243, Korea; inhaek@jejunu.ac.kr; 2 Research Institute of Obesity Sciences, Sungshin Women’s University, Seoul 01133, Korea; mypinehill@naver.com; 3 Department of Food and Nutrition, Seoul Women’s University, Seoul 01797, Korea; sjyang89@swu.ac.kr; 4 Department of Food and Nutrition, Sungshin Women’s University, Seoul 01133, Korea

**Keywords:** lipoprotein lipase, nordihydroguaiaretic acid, dyslipidemia, HDL particle size

## Abstract

Lipoprotein lipase (LPL) hydrolyzes triglycerides in lipoprotein to supply fatty acids, and its deficiency leads to hypertriglyceridemia, thereby inducing metabolic syndrome (MetSyn). Nordihydroguaiaretic acid (NDGA) has been recently reported to inhibit LPL secretion by endoplasmic reticulum (ER)-Golgi redistribution. However, the role of NDGA on dyslipidemia and MetSyn remains unclear. To address this question, leptin receptor knock out (KO)-db/db mice were randomly assigned to three different groups: A normal AIN76-A diet (CON), a Western diet (WD) and a Western diet with 0.1% NDGA and an LPL inhibitor, (WD+NDGA). All mice were fed for 12 weeks. The LPL inhibition by NDGA was confirmed by measuring the systemic LPL mass and adipose LPL gene expression. We investigated whether the LPL inhibition by NDGA alters the metabolic phenotypes. NDGA led to hyperglycemia, hypertriglyceridemia, and hypercholesterolemia. More strikingly, the supplementation of NDGA increased the percentage of high density lipoprotein (HDL)_small_ (HDL_3a+3b+3c_) and decreased the percentage of HDL_large_ (HDL_2a+2b_) compared to the WD group, which indicates that LPL inhibition modulates HDL subclasses. was NDGA increased adipose inflammation but had no impact on hepatic stress signals. Taken together, these findings demonstrated that LPL inhibition by NDGA aggravates metabolic parameters and alters HDL particle size.

## 1. Introduction

Dyslipidemia is characterized by high levels of total cholesterol (TC), low-density lipoprotein (LDL) cholesterol, triglyceride (TG), and low levels of high density lipoprotein (HDL). It is considered a major risk factor for cardiovascular diseases (CVD) [1,2]. Among the components of dyslipidemia, a high level of LDL cholesterol (LDL-C) (>200mg/dl) is strongly associated with CVD events. However, atherosclerosis is common even in those with normal LDL levels (90–130mg/dl) [3,4]. A recent article also mentioned that LDL-C is not a good predictor of CVD with type 1 diabetes [5]. Furthermore, a systemic review of the effect of hypolipidemic agents on mortality showed that only 30% of atherosclerosis can be accounted for by LDL cholesterol, and that other factors, beyond the LDL cholesterol concentrations, are closely involved in the development of atherosclerosis [5,6]. This might indicate that LDL cholesterol may not discriminate between those who develop CVD and those who do not. HDL cholesterol has been considered a protective factor for lipid-related diseases due to its transport of excess cholesterol from peripheral tissues to the liver, as well as its inhibition of LDL cholesterol oxidation and inflammation [7]. Observations from epidemiological studies suggested that HDL cholesterol is negatively correlated with the risk of CVD [8], and this is generally accepted in clinical settings. However, recent clinical attempts to raise HDL cholesterol levels reported no or little correlation between HDL cholesterol and CVD [9,10]. A possible explanation for the neutral results is that there are various HDL phenotypes, depending on the specific settings (e.g., body fat content, body mass index, and types of stresses and diseases). HDL subclasses are divided by size and particle distribution; large HDL subclasses (HDL_2a and 2b_) are inversely related to metabolic syndrome and CVD, and small HDL subclasses (HDL_3a, 3b, and 3c_) may be positively associated with the diseases mentioned above [11,12]. HDL particles are continuously being remodeled by the reverse cholesterol transport (RCT) system. Plasma factors—‘such as LPL, hepatic lipase, lecithin-cholesterol acyltransferase (LCAT), cholesteryl ester transfer protein (CETP), and phospholipid transfer proteins, or cell surface components like ATP-binding cassette (ABC), sub-family A, member 1 (ABCA1), ABC, sub-family G (WHITE), member 1 (ABCG1), and scavenger receptor class B, member 1 (SR-B1)—are involved in the alteration of the reverse cholesterol transport (RCT) system [7], which can modulate HDL metabolism. Thus far, limited evidence has been available regarding the interaction between the RCT system and HDL phenotypes. It has been reported that CETP increases small-size HDLs and small LDLs by interacting with LPL, increasing the risk of atherosclerosis [13]. In contrast, CETP inhibition resulted in increased HDL cholesterol, which suggests that CETP is a potential therapeutic target [14,15,16,17]. Similarly, the CETP inhibitor TA-8995 was also reported as causing a dose-dependent increase of HDL-C levels and Apo-A1 levels while reducing LDL-C levels and Apo-B levels [18], but several CETP inhibitors failed in clinical trials due to insufficient efficacy [19,20,21]. With regard to LPL, LPL-deficient human subjects had low levels of HDL cholesterol and hypertriglyceridemia, accompanied by increased levels of acylation-stimulating protein [22,23]. However, the effect of LPL on HDL subclasses has not yet been investigated.

Therefore, in this study, we investigated whether LPL inhibition affects dyslipidemia and HDL particle size distributions in Western diet (WD)-fed db/db mice by analyzing the lipid profile, insulin resistance, and HDL subclasses of the mice.

## 2. Results

### 2.1. LPL Inhibition by NDGA

**Figure 1:**
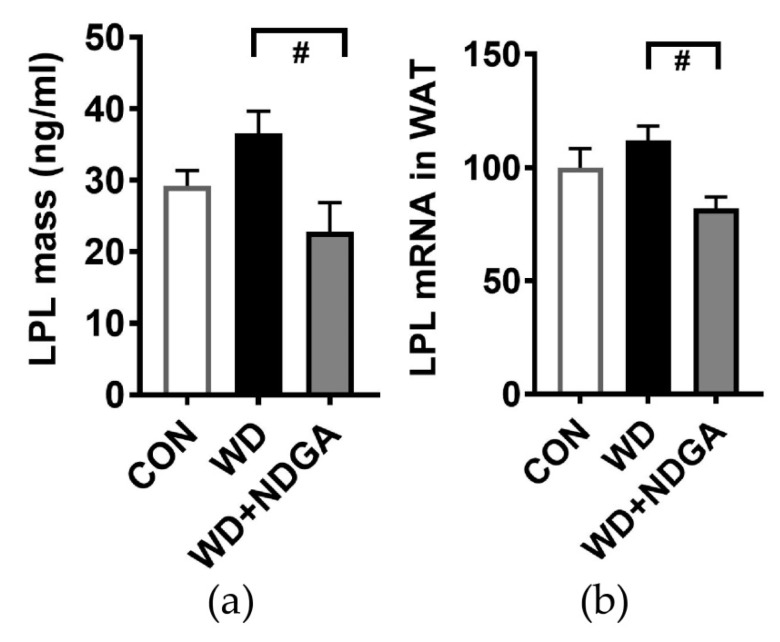
Effects of nordihydroguaiaretic acid (NDGA) on low-density lipoprotein (LPL) mass and LPL gene expression in db/db mice: (**a**) LPL mass by ELISA; (**b**) LPL gene expression by qPCR in adipose tissue. Data are represented as the mean ± SEM of three independent experiments. ^#^ *p* < 0.05, compared with the Western diet (WD) by one-way ANOVA, with Tukey’s comparison test. WAT: White adipose tissue.

We first investigated whether nordihydroguaiaretic acid (NDGA) supplementation inhibits LPL levels. Db/db mice were reported to have a lowered LPL mass and LPL gene expression, compared to C57BL/6 mice [24]. In this study, LPL mass and LPL gene expression in adipose tissue were significantly decreased in the WD+NDGA supplemented group compared to the WD group (Figure 1).

### 2.2. Changes of Metabolic Parameters, Glucose and Insulin Levels by LPL Inhibitor, NDGA in db/db Mice

**Figure 2:**
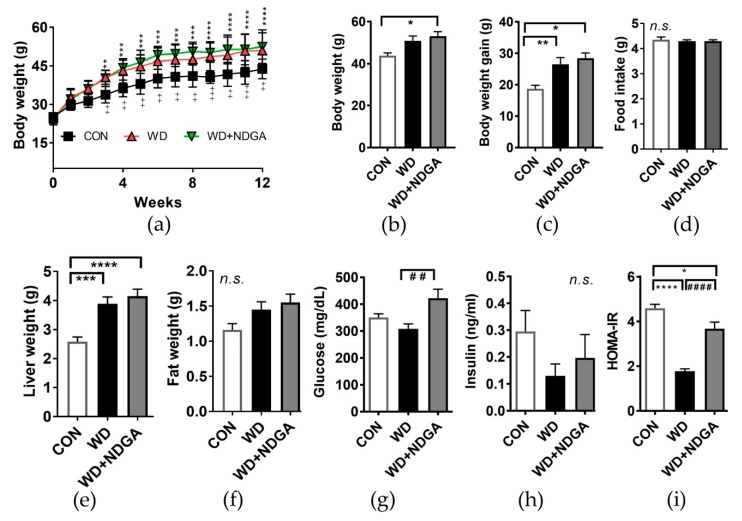
NDGA supplementation altered metabolic parameters, without altering the food intake. Six-week-old male db/db mice were fed with a control (normal AIN76-A (CON), black square, white bar), WD (red triangle, black bar), or WD+NDGA (green triangle, grey bar) diet for 12 weeks (*n* = 10 per group): (**a**) BW, g; (**b**) BW, g; (**c**) BW gain, g; (**d**) food intake, g/day; (**e**) liver weight, g; (**f**) fat weight, g; (**g**) glucose, mg/dl; (**h**) insulin, ng/mL; and (**i**) HOMA-IR. Data are expressed as mean ± SEM (*n* = 10). (**a**) ** *p* < 0.01; *** *p* < 0.001; **** *p* < 0.0001 CON vs WD+NDGA; ^++^ *p* < 0.01; ^+++^ *p* < 0.001; ^++++^ *p* < 0.0001 CON vs WD by two-way ANOVA, with Bonferroni’s multiple comparisons test; (**b**–**i**) * *p* < 0.05; ** *p* < 0.01; *** *p* < 0.001; **** *p* < 0.0001, compared with CON; ^##^ *p* < 0.01; ^####^ *p* < 0.0001, compared with WD by one-way ANOVA, with Tukey’s comparison test.

Next, we investigated whether NDGA supplementation alters metabolic parameters. Mice fed with a WD which mimics human type-2 diabetes [25] significantly promoted body weight (BW) gain, compared to mice fed with a normal AIN76-A (CON) diet after 12 weeks of the diet (Figure 2A–C). NDGA supplementation was partially exacerbated BW and BW gain compared to the WD, without altering the food intake (Figure 2A–D). A similar trend was found in liver and visceral fat (epididymal) mass (Figure 2E,F).

We then asked whether NDGA aggravates type-2 diabetes-mediated abnormal glucose metabolism. The inclusion of NDGA led to an abnormal increase of blood glucose concentration, compared to the levels for the WD group (Figure 2G). While insulin levels had no difference in the NDGA group, compared to the WD group, the homeostasis model assessment of insulin resistance (HOMA-IR) index, an indicator of insulin resistance, was significantly increased by NDGA, compared to the WD group (Figure 2H,I). These data indicated that NDGA exacerbated the type-2 diabetes-induced glucose and insulin intolerance in db/db mice.

### 2.3. Changes of Lipid Profiles and HDL Particle Size by the LPL Inhibitor, NDGA, in db/db Mice

**Figure 3:**
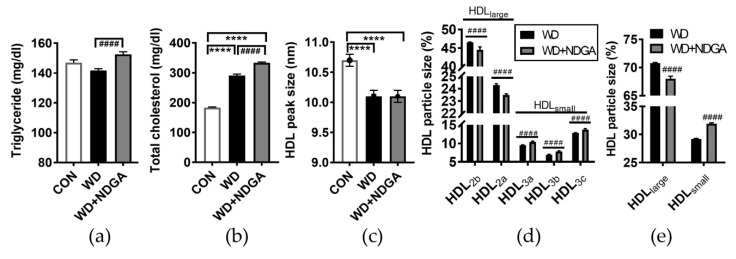
NDGA supplementation altered lipid profiles and high-density lipoprotein (HDL) subclasses. Six-week-old male db/db mice were fed with the control (CON, white bar), WD (black bar), or WD+NDGA (grey bar) diet for 12 weeks (*n* = 10 per group): (**a**) Triglyceride, mg/dl; (**b**) total cholesterol, mg/dl; (**c**) HDL peak size, nm; (**d**) HDL particle size distribution, %; (**e**) HDL particle size distribution, %, sum of HDL2 as expressed HDL_large_, sum of HDL3 as expressed HDL_small_. Data are expressed as mean ± SEM (*n* = 10). **** *p* < 0.0001, compared with the CON ^####^ *p* < 0.0001, compared with the WD by one-way ANOVA, with Tukey’s comparison test.

We postulated that the inclusion of NDGA in the WD diet affects the plasma lipid profile. To address this hypothesis, the levels of the TG and total cholesterol were measured in plasma. TG and total cholesterol were even more up-regulated by NDGA compared to the WD control (Figure 3A,B).

Recently, MetSyn and ischemic stroke were reported to link small HDL3 and reduce large HDL2 levels [26,27]. We next were wondering whether NDGA affects HDL particle size in WD-fed db/db mice. The HDL peak size was decreased in both the WD and WD+NDGA groups compared to the WD group (Figure 3C). Furthermore, NDGA exerted profound effects on HDL particle size by damping the portions of HDL_large_ (HDL_2a+2b_) and increasing the portions of HDL_small_ (HDL_3a+3b+3c_), compared to the WD group (Figure 3D). These data demonstrated that supplementation with the LPL inhibitor, NDGA, leads to dyslipidemia and alters HDL subpopulations which are associated with MetSyn and CVD risk.

### 2.4. Hepatic Stress Levels by the LPL Inhibitor, NDGA, in db/db Mice

**Figure 4:**
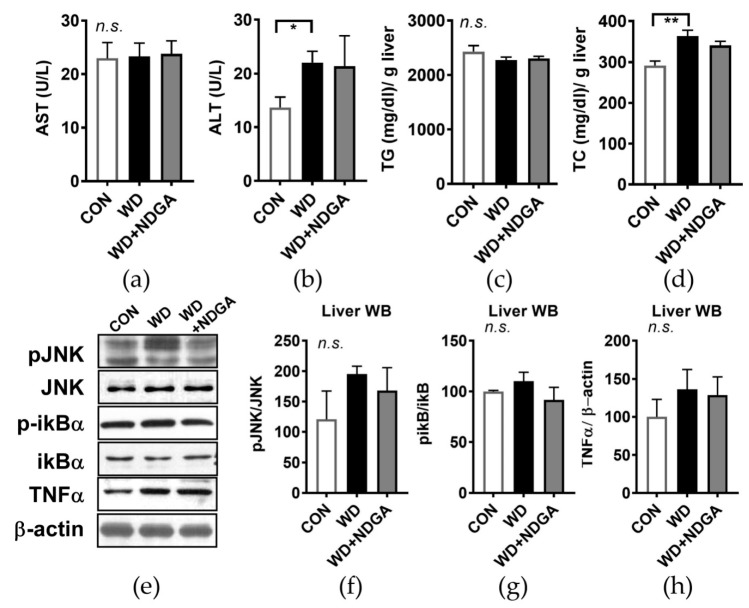
NDGA supplementation did not alter hepatic stress signaling. Six-week-old male db/db mice were fed with a control (CON, white bar), WD (black bar), or WD+NDGA (grey bar) diet for 12 weeks (*n* = 10 per group): (**a**) aspartate aminotransferase (AST) U/L; (**b**) alanine aminotransferase (ALT), U/L; (**c**) triglyceride (TG) contents in liver, mg/dl/g liver; (**d**) total cholesterol (TC) contents in the liver, mg/dl/g; (**e**) hepatic western blot analysis of phosphorylation of c-Jun N-terminal kinases (JNK), phosphorylation-inhibitor kappa B (p-ikB), and tumor necrosis factor alpha (TNFα) in CON-, WD-, or WD+NDGA-fed db/db mice; (**f**) triplicate of western blot analysis of pJNK/JNK; (**g**) triplicate of western blot analysis of p-iKB/iKB; and (**h**) triplicate of western blot analysis of TNFα/β-actin. Data are expressed as the mean ± SEM (*n* = 10). * *p* < 0.05; ** *p* < 0.01, compared with the CON, by one-way ANOVA, with Tukey’s comparison test.

Given the significant alteration of plasma lipid and glucose profile by NDGA (Figure 2 and Figure 3), we hypothesized that NDGA supplementation causes hepatic steatosis and endoplasmic reticulum (ER) stress. There was no difference in the aspartate aminotransferase (AST) and alanine aminotransferase (ALT) levels, which are known as systemic indicators of liver damage, between the WD and WD+NDGA groups (Figure 4A,B). Next, we investigated whether NDGA leads to a fatty liver by measuring the TG and cholesterol content in hepatic tissue. No differences were found in lipid contents between the NDGA-fed group and WD-fed group (Figure 4C,D). It is well known that hepatic ER stress is associated with hepatic steatosis [28], so we investigated whether NDGA altered hepatic stress signals. The WD diet-fed mice showed a marginal, not significant increase in c-Jun N-terminal kinases (JNK) activation by the induction of the phosphorylation of the JNK (pJNK) expression, compared with the CON group. However, there were no differences between NDGA and the WD (Figure 4E,F). Furthermore, pro-inflammatory markers, such as the phosphorylation-inhibitor kappa B (p-ikB)/ikB ratio and tumor necrosis factor alpha (TNFα) expression were not significantly different between NDGA and the WD (Figure 4E,G,H). These data indicated that hepatic lipid accumulation and oxidative stress signals were not affected by the LPL inhibitor, NDGA.

### 2.5. LPL Inhibition Alters Inflammation-Relation Makers in Epididymal Fat

**Figure 5:**
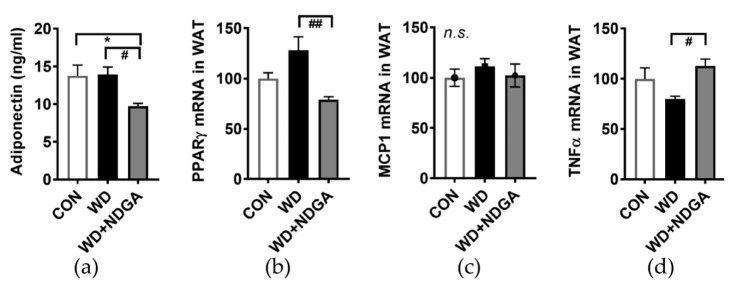
NDGA supplementation increased adipose inflammatory expression. Six-week-old male db/db mice were fed with a control (CON, white bar), WD (black bar), or WD+NDGA (grey bar) diet for 12 weeks (*n* = 8−12 per group): (**a**) Serum adiponectin (ng/mL); (**b**) mRNA expression levels of proliferator-activated receptor gamma (PPARγ); (**c**) MCP1; and (**d**) TNFα in epididymal adipose tissue, quantified by qPCR. Data are expressed as mean ± SEM (*n* = 8–12). * *p* < 0.05, compared with the CON; ^#^ *p* < 0.05, ^##^ *p* < 0.01 by one-way ANOVA, with Tukey’s comparison test.

To determine whether the inclusion of NDGA alters adipose tissue metabolism and adipose inflammation, serum adiponectin levels and several inflammatory gene expressions were measured in epididymal fat. A marginal increase of fat mass (Figure 2) was associated with lowered plasma levels of adiponectin levels in the WD+NDGA group, compared to the WD group (Figure 5A). Consistently, the adipocyte-transcription factor, peroxisome proliferator-activated receptor gamma (PPARγ), was also down-regulated by NDGA, compared to the WD group (Figure 5B). Despite there being no differences in monocyte chemoattractant protein-1 (MCP-1/CCL2) expression (Figure 5C)—which is one of the key chemokines that regulate the migration and infiltration of monocytes/macrophages, in the NDGA group—pro-inflammatory gene expressions, such as TNFα, were increased by NDGA in epididymal adipose tissue (Figure 5D). This suggests that LPL inhibition increased inflammation in adipose tissue.

## 3. Discussion

LPL is a multifunctional enzyme produced by several tissues, such as adipose tissue, cardiac and skeletal muscle, and pancreatic tissue. LPL hydrolyzes the TG core of TG-rich lipoproteins, including chylomicrons, and very low-density lipoproteins (VLDL) as a rate-limiting enzyme [29]. Human subjects with LPL deficiency showed low HDL cholesterol and hypertriglyceridemia [22,23]. LPL deficiency had consistent phenotypes in not only in a human but also in rodent models. However, the effect of LPL inhibition on insulin resistance (IR) and lipid metabolism remains ambiguous. This study was specifically designed to test the hypothesis that the inclusion of NDGA, the functional role of which is to be an LPL inhibitor, exacerbates metabolic complications and alters HDL subclasses in the type-2 diabetes mice model, db/db mice. Here, we demonstrated that the addition of NDGA in a WD marginally impedes metabolic parameters (Figure 2), alters lipid profiles (including HDL particle size (Figure 3) and systemic glucose levels (Figure 2)), and up-regulates adipose inflammation (Figure 5), all without changing hepatic stress signals (Figure 4) in db/db mice. To our knowledge, this is the first study to report the previously unrecognized function of NDGA as an LPL inhibitor in the augmentation of metabolic dysfunction in db/db mice and its possible association with the alteration of HDL subclass distribution.

The major distinctive metabolic consequence that we first noticed was that NDGA differentially impacted metabolic parameters in db/db mice. Db/db mice are well known type-2 diabetes-animal models and showed a significant increase in BW after four weeks of being fed a standard diet [30]. Consistently, our data also showed that db/db mice had a continuous increase of BW. A WD did stimulate BW up-regulation compared to CON. However, the additional supplementation of NDGA did not impede metabolic parameters such as BW or organ weight compared to a WD (Figure 2 A,B), which may be due to its plateau of db/db mice. Interestingly, NDGA caused hyperglycemia and the induction of HOMA-IR levels (Figure 2G–I). In agreement with this notion, Laperrousaz et al. recently reported that neuronal LPL deficiency causes body weight gain and induces the early dysfunction of glucose homeostasis [31]. However, the phenotypes of LPL deficiency may vary, depending on the tissue. The adipose tissue-specific LPL deficiency model, unlike the neuronal LPL deficiency model, showed no difference in body weight but did show hypertriglyceridemia [32]. Strikingly, on the other hand, mice with an over-expression of adipose tissue-specific LPL and fed with high-fat diet had improved glucose intolerance but no difference in body weight [33]. This might indicate the pivotal role of LPL on glucose and lipid metabolism. NDGA has been reported to inhibit LPL secretion by ER-Golgi redistribution [34], but it is still unclear how much and what types of tissue are affected by NDGA. Though we measured LPL mass and LPL gene expression in adipose tissue (Figure 1), further study is necessary to determine the dose-duration LPL activity caused by NDGA in different tissues. Moreover, we have used db/db mice fed with a WD as our animal model, but we did not check the impact of NDGA on control mice strains such as C57BL/6J. Future investigations are required to test this hypothesis.

NDGA supplementation leads to dyslipidemia, which is evidenced by an increase in plasma TG and TC and a decrease in the HDL peak size, as well as HDL subclass distributions (Figure 3). In agreement with this notion, Murase et al. reported that the inhibition of LPL caused hypertriglyceridemia in humans [35], and Berbee et al. reported that human APOC1 transgenic mice, who had a down-regulated LPL, showed hypertriglyceridemia and hypercholesterolemia [36]. A reduced HDL2, which is large HDL, is associated with angiographically-defined arteriosclerosis [37,38]. In our study, NDGA supplementation in a WD caused a significant reduction of HDL2 and the induction of HDL3 (Figure 3). This indicated that the modulation of the RCT enzyme is able to regulate HDL particle size. We expected that the complexities of HDL metabolism might be explained by the relation between the RCT system and HDL particle size distribution. The LPL inhibition increased the proportion of HDL_small_ (HDL_3a+3b+3c_), which is related to the risk of metabolic syndrome [11,12]. Accordingly, the protective effect of HDL_large_ (HDL_2a+2b_) against metabolic syndrome was attenuated by LPL inhibition, strengthening the effect of HDL_small_ (HDL_3a+3b+3c_) on metabolic complications. Furthermore, an increased plasma TC caused by LPL inhibition may be due to increased levels of circulated LDL and/or an altered exchange of cholesterol ester-rich HDL. VLDL-TG may be exchanged to HDL, resulting in decreased levels of VLDL-TG due to LPL inhibition. While we cannot rule out the significant effects of other regulating factors of the RCT system, it is clear that, in the setting of obesity and hyperlipidemia, LPL inhibition-mediated alterations are associated with an increased risk of metabolic complications. Moreover, the findings in this study suggest that LPL is highly associated with HDL subclasses. Different HDL properties have been reported to associate with several diseases such as ischemic stroke [27], CVD [12], and hyperglycemia [39]. Our previous study also revealed that a high level of HDL3 is associated with large waist circumference and MetSyn [40]. In agreement of this notion, a recent paper reported the association between Metsyn and HDL particle size [26,41]. Several studies have shown that HDL3 exerts a more powerful protective action against LDL oxidation than HDL2 [42,43]. However, the effect of the individual HDL subfraction on the risk of diseases is still ambiguous. We did not check detailed lipoprotein profiles (such as VLDL, LDL, and HDL levels), the clearance of TG, and lipoprotein receptor expressions in the liver or skeletal muscle, which may affect TG metabolisms caused by NDGA, so that aspect needs to be investigated. Moreover, future studies using an LPL agonist alone or in combination with a CETP inhibitor need to be conducted in order to investigate the possibility of LPL as a therapeutic target of dyslipidemia and CVD.

Targeting both liver and adipose tissue on the modulation of lipid accumulation would be the goal for intervention strategies to control MetSyn and obesity-related metabolic complications. The present study was designed to determine the diminished lipid metabolism of NDGA supplementation in adipose and the liver and to identify the metabolic alterations caused by NDGA in both tissues. We expected to find a higher ER stress and hepatic inflammation status due to NDGA, which is evidenced by the increased plasma TG and TC level, as well as HDL_large_. Unexpectedly, hepatic lipid accumulation and hepatic stress signals were not altered by the LPL inhibitor, NDGA (Figure 4). Since many reports demonstrated that hepatic LPL levels are relatively low compared with other tissues [44], little attention has been paid to hepatic lipid metabolism. Recent evidence reported that hepatic TG, cholesterol contents, and/or glucose homeostasis did not change due to hepatic LPL deficiency [45]. Thus, we assumed that NDGA, LPL inhibitor-mediated hypertriglyceridemia, hypercholesterolemia, hypoadiponetimia, and hyperglycemia are compensated due to the action of hepatic lipid homeostasis caused by LPL. However, further study is needed to confirm the hepatic lipid homeostasis action of NDGA in vitro or the control strain of mice.

Lastly, we assessed the impact of NDGA on adiposity as well as adipose tissue inflammation. The fat weight did not reach statistical significance among the groups despite the trend of a stepwise incline in mice fed with WD+NDGA>WD>CON diets (Figure 2). The adiponectin levels were decreased due to LPL inhibition (Figure 5), which may be due to decreased insulin sensitivity. TNFα mediates both apoptosis and inflammation, stimulating an inflammatory cascade through the non-canonical pathway of NF-kappaB activation, leading to an increased nuclear RelB and p52 [46]. TNFα was increased in NDGA-treated mice (Figure 5), suggesting that TNFα-related mechanisms in fat cells, such as the secretion of adipokines (e.g., adiponectin), ER stress, and the disturbance of insulin-signaling pathways were changed by LPL inhibition through NDGA. All of these alterations imply the insulin resistance was aggravated by LPL inhibition. However, we still did not measure the systemic inflammatory cytokines or non-canonical pathway of NF-κB activation in adipose tissue. Further studies should be conducted to validate these hypotheses.

In summary, we tested the innovative idea that the inclusion of NDGA in a WD diet could exacerbate the leptin receptor knock out (KO) mice-mediated metabolic dysfunction. We identified that NDGA, as an LPL inhibitor, effectively worsened obesity and metabolic complications including dyslipidemia, HDL particle size alteration, and adipose inflammation. These initial findings in rodents must be confirmed by dose and duration experiments in rodents and humans in future studies. A major limitation of our study is that it is still hard to identify the HDL phenotype by NDGA, an LPL inhibitor. HDL is a highly heterogeneous lipoprotein class, so further study is required by measure HDL concentration, size, density, shape, apolipoprotein compositions, and complete lipoprotein distribution. We are currently investigating a rodent study by using apolipoprotein E-knockout (apoE KO) mice as our mice model to induce atherosclerosis and identify the role of the LPL inhibitor NDGA on CVD risk and HDL subpopulations. Nevertheless, we believe that our study sheds new insight into how NDGA alters metabolic phenotypes and HDL particle size.

## 4. Materials and Methods

### 4.1. Care of Animals and Experimental Groups

Male wild-type and db/db mice were provided by Japan SLC Inc. (Tokyo, Japan). Thirty db/db mice were maintained in a temperature- and humidity-controlled room on a 12 h light/dark cycle and fed the irradiated standard, American Institute of Nutrition (AIN)-76A. After one week of the acclimation period, 30 db/db mice, at approximately 6 weeks of age, were divided into three experimental groups: 1) db/db mice with a standard diet (AIN-76A diet; CON), 2) db/db mice with a WD (21% milk fat, DYET #102068, Dyets Inc., Bethelehem, PA, USA: WD), and 3) db/db mice with a Western diet and 0.1% NDGA as an LPL inhibitor (WD+NDGA), with unlimited access to food and water (Table 1). NDGA was purchased from Dyets Inc. and delivered as a pre-mixed powder diet. The study protocol conformed to the specifications outlined in the National Institutes of Health’s Guiding Principles for the Care and Use of Laboratory Animals and was approved by the Institutional Animal Care and Use Committee of Sungshin Women’s University (01.05.2008, # SSWU AEC 2008-001).

### 4.2. Blood and Tissue Collection

After 12 weeks of treatment, mice, fasted overnight, were anesthetized with diethyl ether (Duksan, Seoul, South Korea), and blood was collected from the carotid artery. After blood collection, the tissues were harvested, weighed, and stored at −80 °C until further analysis.

### 4.3. Measurements of Biochemical Parameters

Plasma glucose (QuantiChrom Glucose Assay Kit, Hayward, CA, USA), TG, TC (Boeringer mannheim, Germany), aspartate aminotransferase (AST), and the alanine aminotransferase (ALT) assay (IFCC, Human 12021, Wiesbaden, Germany) were measured by an absorbance reader (Multiskan spectrum, Thermo Scientific, Waltham, MA, USA). Commercially available ELISA kits were used for the measurement of plasma adiponectin (AdipoGen, Incheon, Korea) and serum insulin (Mercodia AB, Uppsala, Sweden).

### 4.4. HDL Particle Size Phenotyping

HDL particle size phenotypes were determined as previously described [40]. The HDL fraction was isolated by the new micro-ultracentrifugation at 1.063 < d < 1.21 g/mL, with a high relative centrifugal field (RCF) of 1,050,000 × *g* for 140 min (Hitachi CS150GXL, S140AT fixed angle rotor, Tokyo, Japan). The final HDL preparations were dialyzed, and 10 μL of HDL, was loaded onto 4–30% polyacrylamide non-denaturating gradient gels (LPE gels 4/30, CBS Scientific Company, Inc., Del Mar, CA, USA), which were run by a Pore Gradient Lipoprotein electrophoresis system (PGGE, CSI Scientific Electrophoresis LPE-4003; C.B.S. Scientific Inc., Solana Beach, CA, USA). They were pre-run at 80 Voltage for 20 min with a non-SDS buffer, and the voltage was applied in the following way: 100 V for 2 h, 130 Voltage for 4 h, 150 Voltage for 18 h, and 120 Voltage for 2 h. The Coomassie Blue G-250 stained gels were analyzed using the ImageMaster ID software 4.0 (Amersham Pharmacia Biotech, Buckinghamshire, UK). The diameter of the lipoprotein band was calibrated by computing a log-linear standard curve with high molecular weight calibration kit standards (thyroglobulin, 17 nm; ferritin, 12.2 nm; catalase, 10.4 nm; LDH, 8.2 nm; BSA, 7.1 nm; Amersham Pharmacia Biotech) as the relative migration distances.

### 4.5. Preheparin LPL Mass

The preheparin LPL mass was measured using ELISA kits (LPL ELISA kit, Daiichi Pure Chemicals, Tokyo, Japan), according to the manufacturer’s protocol.

### 4.6. Gene Expression Analysis (Quantitative PCR)

The total RNA was isolated by a Trizol reagent (Invitrogen, Carlsbad, CA, USA). The RNA was subsequently reverse-transcribed to cDNA using the ThermoScript RT-PCR system (Invitrogen), following the manufacturer’s instructions. The mRNA levels were measured by real-time PCR using a Mini Opiticon (Bio-Rad Laboratories, Hercules, CA, USA). PCR primers for the target genes were designed using an online version of the primer 3 software. Quantitative PCR data were evaluated using the comparative critical threshold (*C*t) method and normalized to the average of the endogenous control gene, β-actin (primer sequences are shown in Table 2). The relative gene expression was calculated by the delta Ct method.

### 4.7. Western Blotting Analysis

The liver was collected, and the protein was isolated with an ice-cold radioimmune precipitation assay (RIPA) buffer (Thermo Scientific) containing protease inhibitors (Sigma). Proteins were fractionated using 8% or 10% SDS-PAGE, transferred to polyvinylidene difluoride (PVDF) membranes and incubated with the relevant antibodies. Chemiluminescence from the enhanced chemiluminescence (ECL) (Western Lightning) solution was detected with ChemiDoc (Bio-Rad). Polyclonal or monoclonal antibodies which target phosphor- c-Jun N-terminal kinases (JNK), JNK, phosphor- inhibitor kappa B (p-ikB), ikB, and β-actin were purchased from Cell Signaling Technology.

### 4.8. Statistical Analysis

All the data were expressed as the means and standard error (SE), and statistical calculations were performed using the t-test and ANOVA (one-way analysis of variance), with Tukey’s and Bonferroni’s multiple comparison tests. The results were considered significant if *p* < 0.05 (GraphPad Prism Version 7.0, La Jolla, CA, USA).

## Figures and Tables

**Table 1:** Diet composition.

	AIN76-A Diet (CON)	Western Diet (WD)	0.1% NDGA Mixed Western Diet (WD + NDGA)
Ingredient	grams/ Kg	grams/ Kg	grams/ Kg
Casein	200	195	195
DL-Methionine	3	3	3
Cornstarch	150	150	150
Sucrose	499.99	341.46	340.46
Cellulose	50	50	50
Milk Fat	50 (corn-oil)	210	210
Cholesterol		1.5	1.5
Vitamin Mix #310035	12	10	10
Mineral Mix #200000	35	35	35
Calcium Carbonate		4	4
Ethoxyquin	0.01	0.04	0.04
NDGA			1

**Table 2:** Primer sequences for qPCR.

Gene	Forward/Reverse	Sequence (5′-3′)
mPPARγ	Forward	aac tgc agg gtg aaa ctc tgg gag att ctc c
	Reverse	gga ttc agc aac cat tgg gtc agc tct
mFAT/CD36	Forward	tac ctg gga gtt ggc gag
	Reverse	ttg cca cgt cat ctg ggt tt
mTNFα	Forward	ctg agg tca atc tgc cca agt ac
	Reverse	ctt cac aga gca atg act cca aag
mβ-actin	Forward	cct cta tgc caa cac agt gc
	Reverse	gta ctc ctg ctt gct gat cc

## Abbreviations

ABCA1	ATP-binding cassette (ABC), sub-family A, member 1
ABCG1	ABC, sub-family G, member 1
ALT	Alanine aminotransferase
AST	Aspartate aminotransferase
CETP	Cholesteryl ester transfer protein
CON	Control, AIN76-A diet
CVD	Cardiovascular diseases
ER	Endoplasmic reticulum
HDL	High-density lipoprotein
IkB	Inhibitor kappa B
JNK	C-Jun N-terminal kinases
LDL	Low-density lipoprotein
LPL	Lipoprotein lipase
MCP1	Monocyte chemoattractant protein-1
MetSyn	Metabolic syndrome
NDGA	Nordihydroguaiaretic acid
PPARg	Peroxisome proliferator-activated receptor gamma
RCT	Reverse cholesterol transport
SR-B1	Scavenger receptor class B, member 1
TC	Total cholesterol
TG	Triglyceride
TNFα	Tumor necrosis factor alpha
WD	Western diet
